# Natsukashii

**DOI:** 10.1192/j.eurpsy.2022.1021

**Published:** 2022-09-01

**Authors:** M.J. Gordillo Montaño, S.V. Boned Torres, L. Rodriguez Rodriguez, M. De Amuedo Rincon

**Affiliations:** Hospital Can Misses, Psychiatry, Eivissa, Spain

**Keywords:** Psychopathology, bipolar disorder, NOSTALGY, euthymia

## Abstract

**Introduction:**

NATSUKASHII: Japanese word that means happy nostalgia, it is the moment in which memory transports you to a beautiful memory that fills you with sweetness. NOSTALGY: (from the classical Greek [nóstos], “return”, and [algos], “pain”) feeling of sadness, suffering of thinking about something that has been had or lived in a stage and now not. In bipolar disorder, patients are more likely to complain of dysphoria than euphoria. Hypomanic periods often provide pleasant relief from depression. Patients experience this situation as pleasant, positive and longing once it has remitted, since they feel more creative, active and sociable.

**Objectives:**

We intend to draw attention to the blurred limits of the state of euthymia, even when stable there is a sustained emotional hypersensitivity, which must be learned to identify and coexist. Behind the desire to be euthymic, in certain patients there is a desire to remain hypomanic and / or manic due to the fact that they have tasted absolute happiness.

**Methods:**

After several interviews with stable patients, we have realized that a great majority want to re-experience the sensations of a hypomanic episode.

**Results:**

After a bibliographic search we have realized that in the West there is no term in psychopathology that describes that longing that they verbalize as “maniac lives happier”

**Conclusions:**

Special attention must be paid to these patients since they have less adherence to treatment and risk of abandoning it.

**Disclosure:**

No significant relationships.

